# Supplementary material for: Comparative multiomics analysis of cell physiological state after culture in a basket bioreactor
Source: Sci Rep. 2022 Nov 23;12:20161. doi: 10.1038/s41598-022-24687-4 (PMC9686226; doi:10.1038/s41598-022-24687-4)
Supplement: Supplementary file 1 — Supplementary Information 1. [file 41598_2022_24687_MOESM1_ESM.zip › raw data/Transcriptomic raw data/6.Enrichment/2.KEGG/TE1vsTC1/ALL/src/csab05414.html]

csab05414


- csab:103217090

- Up regulated genes

103217090(1.81328286766627)

- csab:103243277

- Up regulated genes

103243277(1.88743299439371)

- csab:103219220

- Down regulated genes

103219220(-1.74486921304872)

Close
